# Cell Viability and Immune Response to Low Concentrations of Nickel and Cadmium: An In Vitro Model

**DOI:** 10.3390/ijerph17249218

**Published:** 2020-12-09

**Authors:** Ahra Kim, SangJin Park, Joo Hyun Sung

**Affiliations:** 1 Department of Biology, Institute of Health Sciences, College of Medicine, Gyeongsang National University, Jinju 52727, Korea; ar0704@gnu.ac.kr; 2 Department of Occupational and Environmental Medicine, Soonchunhyang University Hospital Cheonan, College of Medicine, University of Soonchunhyang, Cheonan 31151, Korea; 96460@schmc.ac.kr; 3 Department of Occupational and Environmental Medicine, Institute of Health Sciences, Gyeongsang National University Changwon Hospital, College of Medicine, Gyeongsang National University, Jinju 52727, Korea

**Keywords:** cadmium, cell survival, cytokines, environment, immune, metal, nickel

## Abstract

Environmental exposure to low concentrations of heavy metals is common in the general population, but the toxicity, immune response mechanisms, and the effects of single and mixed metal exposures have not been clearly identified. In this study, A549 cells and Raw264.7 cells were exposed to low concentrations of the heavy metals nickel (Ni) and cadmium (Cd) for 24, 48, and 72 h, and then cell viability and cytokine levels were analyzed. We found that exposure to low concentrations of Ni (50 nM) or Cd (10 nM) alone did not affect cell viability. However, mixing them together decreased cell viability. In addition, the levels of IL-10, IL-12, and TNF-α decreased with single (only Cd) and mixed (Ni and Cd) exposures. These results show that exposure to low concentrations of heavy metals could affect the normal immune response, even without obvious clinical manifestations. Therefore, chronic exposure to heavy metals might have adverse effects on overall health.

## 1. Introduction

The effects of heavy metals on the human body are well studied and have identified the target organs and clinical symptoms of heavy metals known to be harmful to humans [1,2]. In addition, toxicokinetic studies of heavy metals have provided information on their absorption pathways, distribution, metabolism, and excretion [3], as well as reported the concentrations that adversely affect health [4]. These results are applied to limit human exposure and set environmental standards in the workplace. These studies provide guidance in limiting human exposure, setting environmental standards in the workplace, and preventing heavy metal poisoning in humans. As a result, occupational diseases caused by common heavy metal poisoning have been reduced in Korea [5].

However, people continue to be exposed to low levels of heavy metals in their surroundings. The Korean National Health and Nutrition Examination Survey (KNHANES) found heavy metals in blood and urine samples from the general population [6], and that the heavy metals of a parent had an effect on a fetus [7]. However, there is insufficient research on the mechanisms by which low concentrations of environmental heavy metals effect the body.

In general, there are many types of heavy metals that can contribute to environmental exposure, especially nickel (Ni) and cadmium (Cd), which have cellular and immune system toxicity [8,9]. Previous studies have shown that cell viability decreases, and inflammatory cytokines increases, in response to Ni and Cd exposure [10,11,12,13,14,15]. However, due to the nature of environmental exposure, it is possible to be exposed to lower concentrations than reported, and there is a high risk of chronic or mixed exposure. Nevertheless, studies on the effects of low concentrations of Ni and Cd at the cellular level and mixed exposure are limited.

In this study, we selected Ni and Cd as representative heavy metals to test environmental exposure and determine the concentration at which a single exposure cytotoxicity. In addition, we evaluated the cytotoxicity and immune responses that appeared with single or multiple exposures to investigate possible mechanisms of their effects.

## 2. Materials and Methods

### 2.1. Cell Culture

The human lung cancer A549 cell and the murine macrophage Raw264.7 cell were obtained from Virus Laboratory of the Ulsan University. Cultures of the A549 and Raw264.7 cell lines were maintained in RPMI 1640 or DMEM media (Gibco, CA, USA) supplemented with 10% fetal bovine serum (FBS, Gibco, CA, USA), 1% penicillin-streptomycin solution, 1% l-glutamine, and 1% nonessential amino acids and cultured at 37 °C and 5% CO_2_, respectively.

### 2.2. Cell Viability Assay

The cytotoxicity of heavy metals was measured using a Cell Counting Kit-8 (CCK-8) assay (Dojindo Laboratories, Kumamoto, Japan). A549 cells seeded in 24-well plates at a density of 2 × 10^4^/mL were exposed to 10 nM, 20 nM, 50 nM, 100 nM, 2 µM, and 10 µM of Ni and Cd and then incubated for 24, 48, and 72 h at 37 °C and 5% CO_2_. Metal salts of NiO and CdCl_2_ were purchased from Sigma Chemical Co. (St. Louis, MO, USA). The control was identical to the test samples, except for the addition of Ni and Cd. The absorbance of control and test samples was quantified at 450 nm using a microplate reader (SpectraMax iD3, Molecular Devices, Shanghai, China). All groups were tested independently over time and were tested in duplicates at least 5 times.

### 2.3. Enzyme Linked Immunosorbent Assay (ELISA)

The release of cytokines was measured using an ELISA kit (R&D Systems Inc., Minneapolis, MN, USA). Raw264.7 cells were seeded in 24-well plates at a density of 2 × 10^4^/mL and treated with Ni (50 nM), Cd (10 nM), and a mixture of Ni and Cd, and then incubated for 24, 48, and 72 h at 37 °C and 5% CO_2_. The control was identical to the test samples, except for the addition of heavy metals. The concentration of cytokines (IL-4, IL-10, IL-12, IL-13, INF-γ, and TNF-α) was measured in the supernatant. The absorbance of control and test samples was quantified at 450 nm using a microplate reader (SpectraMax iD3, Molecular Devices, Shanghai, China). All groups were tested independently over time and were tested in duplicates at least 5 times.

### 2.4. Statistical Analysis

All data are presented as mean ± standard deviation unless otherwise specified. All groups were conducted as independent experiments. Significance was determined by one-way analysis of variance (ANOVA) followed by Tukey’s multiple comparison test for post-hoc comparisons in SPSS 24.0 (IBM Corp., Armonk, NY, USA), and *p*-values lower than 0.05 were considered statistically significant.

## 3. Results

### 3.1. Cell Viability after Heavy Metal Exposure

**Figure 1:**
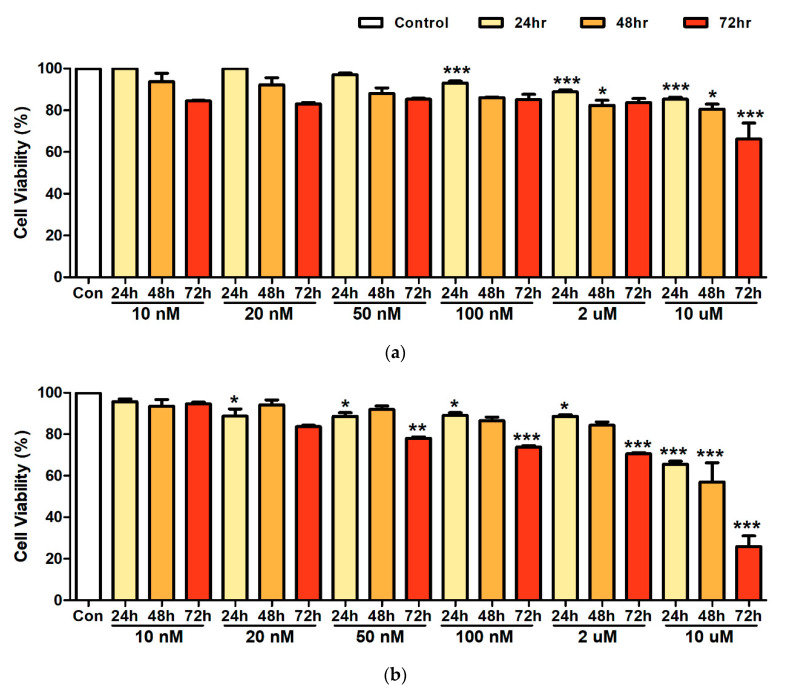
A594 lung cancer cell viability after exposure to Nickel (**a**), and Cadmium (**b**) at concentrations 10 nM, 20 nM, 50 nM, 100 nM, 2 µM, and 10 µM. Bars represent means ± standard deviation. *** *p* < 0.001, ** *p* < 0.01, * *p* < 0.05 vs. control.

To assess the effects of low concentration exposure on cell viability, we treated A594 lung cancer cell cultures with Ni and Cd for 24, 48, and 72 h at the following concentrations: 10 nM, 20 nM, 50 nM, 100 nM, 2 µM, and 10 µM. We found that at concentrations 10–50 nM of Ni, cell viability was not significantly different from that of the control group at any of the time points. However, cell viability was significantly lower than that of the control group after 24 h in cells treated with 100 nM, after 24 and 48 h in cells treated with 2 µM, and all time points in cells treated with 10 µM of Ni. There was no significant difference in cell viability from that of the control group at concentrations of 10 nM of Cd. However, cell viability was significantly lower at 24 h when treated with 20 nM; after 24 and 72 h with 50 nM–2 µM, and all time points when treated with 10 µM of Cd in comparison with the control (Figure 1). Therefore, the highest concentration that did not affect cell viability was 50 nM of Ni and 10 nM of Cd.

**Figure 2:**
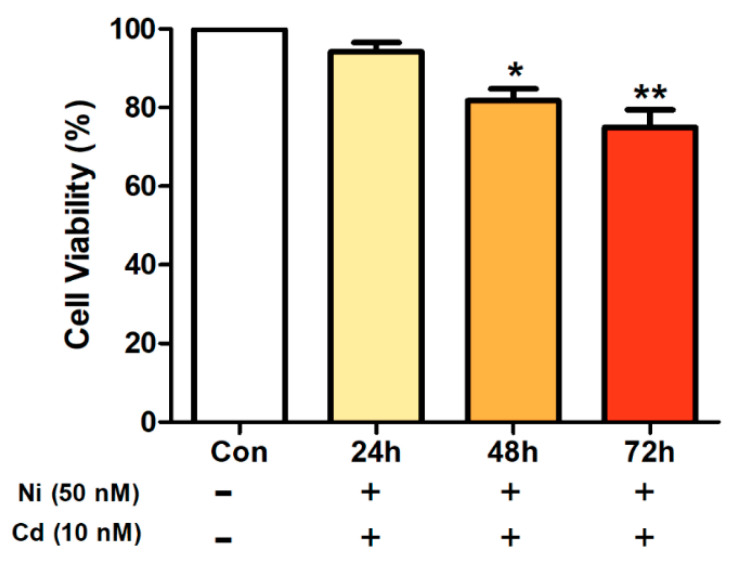
A594 lung cancer cell viability after treatment with a mixture of 50 nM of nickel (Ni), and 10 nM of cadmium (Cd). Bars represent means ± standard deviation. ** *p* < 0.01, * *p* < 0.05 vs. control.

When A594 lung cancer cells were exposed to a mixed treatment of Ni and Cd, there was no significant difference in cell viability after 24 h, but a significant difference was found after 48 and 72 h. Moreover, as more time passed, cell viability decreased further in comparison to the control (Figure 2).

### 3.2. Effects of Heavy Metal Exposure on Cytokines Concentration

**Figure 3:**
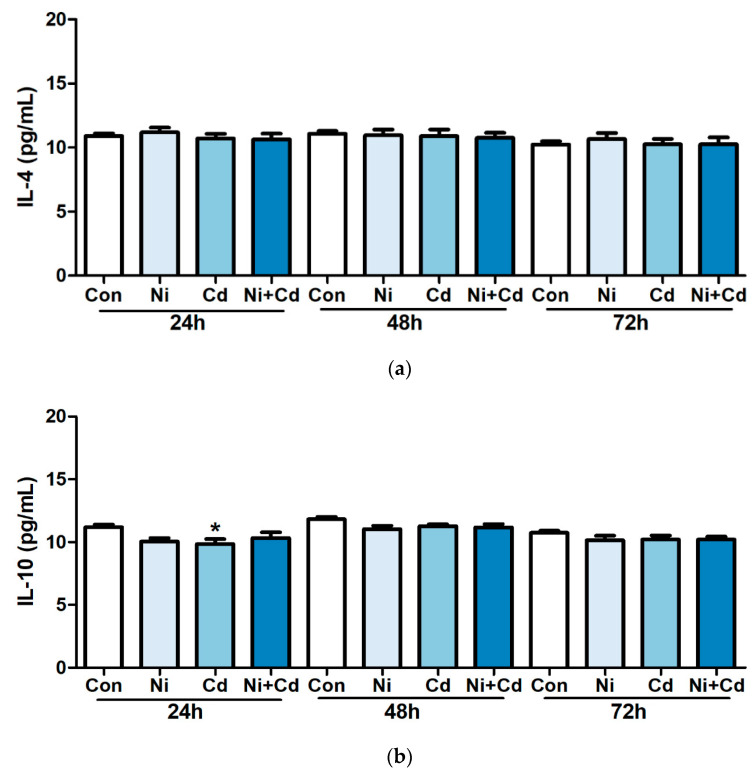

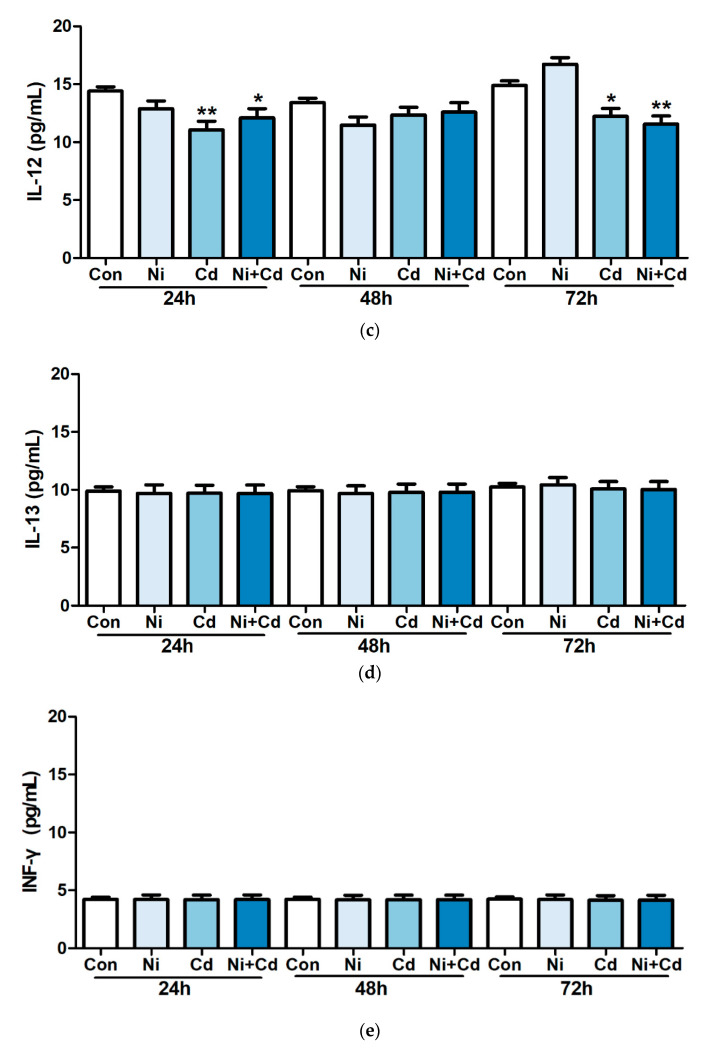

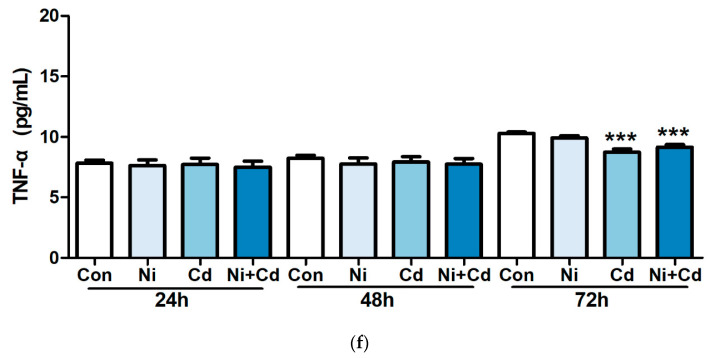
Concentration of cytokines in Raw264.7 murine macrophage cells after exposure to 50 nM of nickel (Ni), 10 nM of cadmium (Cd), and a Ni–Cd mixture. (**a**) IL-4, (**b**) IL-10, (**c**) IL-12, (**d**) IL-13, (**e**) INF-γ, (**f**) TNF-α. Bars represent means ± standard deviation. *** *p* < 0.001, ** *p* < 0.01, * *p* < 0.05 vs. control.

To examine the effect of low-dose exposure on cytokine production, we treated Raw264.7 murine macrophage cell line with 50 nM of Ni, 10 nM of Cd, and a mixture of Ni and Cd for 24, 48, and 72 h. We observed significantly lower levels of IL-10 in cells exposed to Cd alone after 24 h than in the control group. IL-12 was significantly lower in cells exposed to Cd alone and the mixture of Ni and Cd after 24 and 72 h than that in the control group. After 72 h, IL-12 and TNF-α levels were significantly lower in cells treated with mixture of Ni and Cd than in the control group (Figure 3).

## 4. Discussion

In the current study, in order to investigate the cytotoxicity and immune response caused by exposure to low concentrations of the heavy metals, Ni and Cd, which are known to have cytotoxicity and cause immune toxicity [8,9], were selected. Ni generally enters cells through transferrin or divalent metal transporter-1 (DMT1) and exhibits cytotoxicity by inhibiting DNA repair processes or causing oxidative stress through the depletion of intracellular antioxidants such as glutathione [16,17]. Cd enters cells through the ZIP (ZRT, IRT-like protein) transporter, metallothionein, and DMT1 (divalent metal transporter-1). Once in the cell, Cd exhibits cytotoxicity by inhibiting the DNA repair process, cell proliferation and tumor suppressor functions, and causing oxidative stress [18,19,20,21]. Additionally, Cd inactivates the p53 tumor suppressor protein, which is important for cell cycle arrest and apoptosis regulation, thereby inducing DNA damage and inhibiting DNA repair [22].

In order to define the concentrations that have a significant effect on cell viability, A594 lung cancer cells were exposed to Ni and Cd at various concentrations and observed after 24, 48, and 72 h. In addition, in order to confirm the relationship between low-concentration exposure to heavy metals and immune responses, the Raw264.7 murine macrophage cell line was exposed to each heavy metal alone and in mixture, and changes in cytokines were observed. Our results showed that 50 nM of Ni and 10 nM of Cd alone did not have a significant effect on cell viability; however, when the two heavy metals were used in mixture at these concentrations, cell viability was significantly lower than that of the control group after 48 h. Studies on the synergic [23,24] and antagonistic [25,26] effects of heavy metal co-exposure have been conducted, and their results suggest that the mixed exposure of Ni and Cd has a synergic effect on cell viability. Differences in cell entry and cytotoxic mechanisms of Ni and Cd [16,17,18,19,20,21,22] have been identified; therefore, their individual properties may be related to these synergic effects.

We performed cytokine analysis to investigate the immune response to low-concentration heavy metal exposure and several characteristic results were obtained. First, when cells were exposed to Cd alone at 24 h, IL-10, which maintains the balance of the body’s immune system [27], significantly decreased compared with the control. In addition, IL-12, which induces Th1 differentiation and increases INF-γ [28], the mediator of immune response induced by NK cells and T cells, significantly decreased in response to low-concentration heavy metal exposure. Previous studies have reported that when heavy metals are introduced into the body, IL-12 increases first and then INF-γ increases, resulting in a series of immune responses [29,30]. However, in this study, while the exposure to heavy metals decreased IL-12 levels, INF-γ did not change. This result suggests that exposure to low concentrations of heavy metals that do not affect cell viability may inhibit the immune response and promote chronic exposure effects without specific clinical characteristics. Indeed, similar results have been reported in marine organisms in environments where they are chronically exposed to low concentrations of heavy metals [31]. This negative immune response effect may ultimately lead to a condition in which low concentrations of heavy metals result in health effects after chronic, long-term exposure. Therefore, it is necessary to consider these factors when studying the health effects of chronic exposure to low heavy metals in the future.

**Figure 4:**
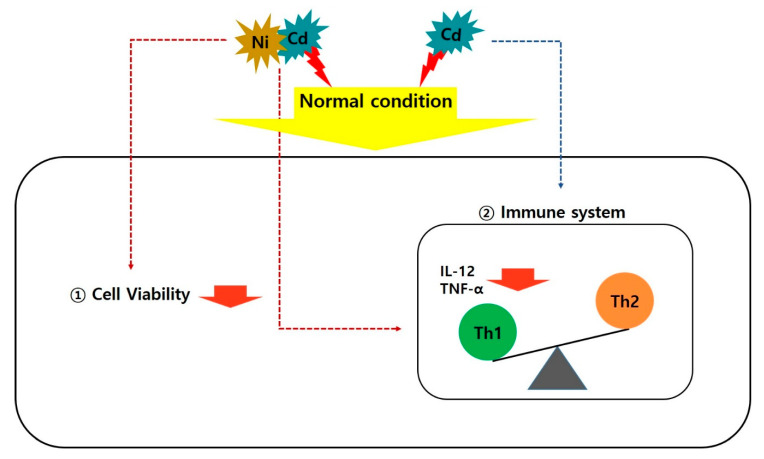
Schematic of cell viability and cytokine production after heavy metal exposure.

Second, we report that IL-12 and TNF-α, which are Th1 cytokines, significantly decreased when exposed to Cd alone and mixture of Ni and Cd after 72 h; however, Th2 cytokines did not change. This is a different result from previous reports of Th1 and Th2 cytokine interactions during a general immune response in which the two types change together [32,33]. Our results suggest that exposure to Cd alone and mixed exposure to Ni and Cd causes a Th2-biased immune response (i.e., decreased Th1 levels but no effect on Th2 levels) and impairs cellular immune responses by inhibiting inflammatory cytokine (TNF-α) secretion by antigen presenting cell (APC) such as macrophages. In other words, chronic exposure to low concentrations of heavy metals alone (Cd) or in complex (Ni with Cd) reduces Th1 cytokines, resulting in an imbalance with the unaffected Th2 cytokines, thereby affecting the overall cellular immune response (Figure 4).

Although this study found meaningful results that further inform of chronic low-level heavy metal exposure, it did have some limitations. First, other cytotoxic heavy metals also affect the immune system, but at this time, other heavy metals were not concomitantly analyzed. Therefore, a more complete dataset could be obtained by assessing other heavy metals in the future. Second, Th1/Th2 cytokines were analyzed using ELISA; however, there are several methods that analyze the immune response that could contribute to accurate determination of the changes and effects between different immune cells. Moreover, up- or downstream activation through mechanistic studies such as qPCR, Western blotting, and in vivo experiments could help further test the hypothesis that immune responses are caused by exposure to low concentrations of heavy metals.

Despite these limitations, this study is unique in its attempt to understand how exposure to very low concentrations (i.e., without any effect on cell viability) of heavy metals could affect the immune response. In addition, the experiments not only tested single heavy metal exposure, but also mixed exposures, providing insight into the potential for synergic effects that could lead to immune responses related to a Th1/Th2 bias. Therefore, we believe that the results of this study present new perspectives on the effects of environmental exposure and low-concentration chronic exposure of various toxic substances, specifically heavy metals, which are currently an environmental issue.

## 5. Conclusions

In this study, in vitro experiments were conducted by selecting representative heavy metals known to cause cytotoxicity and affect the immune system. After determining the concentrations that do not affect cell viability, we investigated their effects on cell viability and cytokine production following single and mixed exposure. Our findings suggest that the mixed exposure to heavy metals exerts a synergic effect on cell viability, particularly because we did not find any effect after single exposure at low concentrations. In addition, we show that both single and complex exposures of low concentrations of heavy metals affect cytokines. Unlike previous studies, although IL-12-mediated immune responses were characteristically decreased, and Th2 cytokines remained unchanged. These results show that exposure to low concentrations of heavy metals results in a characteristic immune response that affects the immune system through the imbalance of Th1/Th2 systems, which is unlike general immune responses in which Th1/Th2 cytokines interact and change together.

These two results have very important implications. Exposure to low concentrations of heavy metals inhibits the normal immune response even though it does not show any specific clinical features. The induced immune response from this persistent low-dose exposure may have adverse health effects that risk going unnoticed by healthcare practitioners. In particular, the implications are even greater with the concerns about increasing environmental diseases. Therefore, when conducting studies on chronic exposure to low concentrations of various hazardous substances, including heavy metals, the experimental design should consider these factors.
